# Prediction of Pseudoprogression versus Progression using Machine Learning Algorithm in Glioblastoma

**DOI:** 10.1038/s41598-018-31007-2

**Published:** 2018-08-21

**Authors:** Bum-Sup Jang, Seung Hyuck Jeon, Il Han Kim, In Ah Kim

**Affiliations:** 1 Department of Radiation Oncology, Seoul National University Hospital, Seoul, Korea; 2 Department of Radiation Oncology, Seoul National University Bundang Hospital, Seongnamsi, Korea; 3 Institute of Radiation Medicine, Cancer Research Institute, Seoul National University College of Medicine, Seoul, Korea

## Abstract

We aimed to investigate the feasibility of machine learning (ML) algorithm to distinguish pseudoprogression (PsPD) from progression (PD) in patients with glioblastoma (GBM). We recruited the patients diagnosed as primary GBM who received gross total resection (GTR) and concurrent chemoradiotherapy in two institutions from April 2010 to April 2017 and presented suspicious contrast-enhanced lesion on brain magnetic resonance imaging (MRI) during follow-up. Patients from two institutions were allocated to training (N = 59) and testing (N = 19) datasets, respectively. We developed a convolutional neural network combined with a long short-term memory ML structure. MRI data, which was 9 axial post-contrast T1-weighted images in our study, and clinical features were incorporated (Model 1). In the testing set, the trained Model 1 resulted in AUC of 0.83, AUPRC of 0.87, and F1-score of 0.74 using optimal threshold. The performance was superior to that of Model 2 (CNN-LSTM model with MRI data alone) and Model 3 (random forest model with clinical feature alone). The developed algorithm involving MRI data and clinical features could help making decision during follow-up of patients with GBM treated with GTR and concurrent CCRT.

## Introduction

Even after the introduction of a standard regimen consisting of concurrent chemoradiotherapy (CCRT) and adjuvant temozolomide, most patients with glioblastoma multiforme (GBM) experience disease progression^1^. Clinicians often encounter a situation where they need to distinguish progressive disease (PD) from pseudoprogression (PsPD) following CCRT. PsPD is resulted from disruption of blood-brain barrier by CCRT and subsequent leakage of contrast material outside blood vessel. The discrimination is challenging because both lesions demonstrate similar contrast enhancement (CE) on gadolinium-enhanced T1-weighted magnetic resonance imaging (MRI)^2,3^. Although pathologic confirmation is the most reliable method to diagnose PD or PsPD, numerous non-invasive attempts have been made for discrimination using diffusion-weighted imaging^4–6^, perfusion imaging^7,8^, or positron emission tomography (PET)^9,10^. While these advanced imaging techniques have shown certain values, most experts do not agree with that traditional MRI such as T1-weighted or T2-weighted MRI can distinguish PsPD from PD^11^. The conventional images, however, may give us a clue when they are analyzed with modern tools such as machine learning (ML).

Recently, ML algorithms are actively employed in the field of oncology. Convolutional neural network (CNN) is one of the ML algorithms that imitates a human visual cortex. CNN is designed to extract the feature maps that are compressed and abstracted from the input images and to perform given tasks with these feature maps. Thus, this model has proved its advantages in pulmonary nodule detection^12^, mitosis detection in microscopic images^13^, and skin cancer classification^14^. As another popular ML algorithm, long short-term memory (LSTM)^15^ was recently introduced to effectively train recurrent neural networks by preventing explosion and vanishment of gradient problems that are common in deep recurrent neural networks^16^. Therefore, LSTM is prominently used in challenging sequence predictions such as automatic image caption generation^17^, automatic translation of text^18^, and automatic handwriting generation^19^. Regarding sequence, combination of CNN with LSTM (CNN-LSTM) were found to predict RNA-protein sequence and structure binding preferences^20^. This architecture has been introduced in visual recognition, image description, and video description^21^.

To the best of our knowledge, there are no studies that investigate the potential role of CNN-LSTM structure in discrimination of PsPD from PD. The specific aim of our study was to demonstrate the feasibility of the ML algorithm in predicting PsPD with conventional images, especially gadolinium-enhanced T1-weighted MRI, in patients with GBM after CCRT.

## Methods

### Patient Selection

The institutional review board at SNUH (Seoul National University Hospital) and SNUBH (Seoul National University Bundang Hospital) approved this study protocol with a waiver of the written informed consent. All methods were performed in accordance with the relevant guidelines and regulations. We retrospectively reviewed patients with primary GBM who underwent gross total resection of enhancing tumor (GTR) followed by CCRT and adjuvant temozolomide from April 2010 to April 2017 at two institutions: SNUH and SNUBH. All patients who exhibited single measurable CE lesion of any size on gadolinium-enhanced T1-weighted MRI within 80% isodose line after CCRT (based on Response Assessment in Neuro-Oncology criteria^22^) were included in the study. The exclusion criteria were as follows: (1) Demonstration of CE lesion on MRI not per institutional protocol, (2) No sufficient follow-up to determine the identity of lesion, (3) Detection of CE lesion before CCRT, (4) Suspicious residual CE lesion at immediate post-operative MRI, indicating incomplete resection, and (5) Incomplete CCRT. Finally, 78 patients (SNUH, N = 59 and SUNBH, N = 19) were included in the present study.

### Data Collection and Preprocessing

In both the institutions, initial and follow-up images were obtained according to the specific protocol for glioma patients. All the images included T1-weighted 3D magnetization-prepared rapid acquisition gradient echo (MPRAGE) sequence before and after administration of gadolinium. Nine successive axial images of post-contrast MPRAGE sequence were selected by clinicians where the fifth image best represents the suspicious CE lesion. MRIs were acquired using 1.5-T (N = 11) or 3-T (N = 67) scanner. The 3D-MPRAGE images were obtained with matrix ranged from 256 × 256 to 1024 × 1024. The median slice thickness was 1-mm (range, 0.86–1.50 mm). Detailed information of imaging parameters is provided in Supplementary Table S1. Because pixel size and field of view (FOV) varied, input images were normalized as follow. First, they were resized into 200 × 200 (mm) images by cropping or padding. This size was selected since FOV was greater than 200 × 200 (mm) in all images but one with FOV of 193 × 193 (mm). The resized images were resampled into 256 × 256 pixels. The intensities of pixels were linearly scaled to have zero mean and unit norm.

The following clinical features were collected from medical records: age at the time of surgery, gender, methylation status of the O6-methylguanine-DNA-methyltransferase (MGMT) promoter, mutational status of the isocitrate dehydrogenase (*IDH*) gene, the total dose and number of fractions of radiotherapy, and the interval between the end of CCRT and the appearance of CE lesion. All clinical parameters were normalized and ranged between 0 and 1.

Forty-eight CE lesions that were surgically confirmed to be PD (N = 20), increased without spontaneous decrease on follow-up MRI (N = 25), or showed significant uptake on PET (N = 3) were classified as PD, and 30 CE lesions that were pathologically proved to be PsPD (N = 3), reduced on follow-up MRI before intervention (N = 21), remained stable for at least 120 days after appearance (N = 5), or no significant uptake on PET (N = 1) were considered as PsPD. The discrimination of lesions in our study was in accord with the multi-disciplinary assessment and treatment planning of the two institutions.

### ML Network Structure

In the present analysis, we utilized the deep CNN-LSTM structure because CNN can learn features from brain MRI and LSTM recognizes the spatial sequence of images. Along with MRI, clinical factors are important when clinicians decide the identity of a lesion. Therefore, clinical parameters including age, gender, total radiation dose, number of fractions, interval between CCRT and appearance of lesion, MGMT methylation status, and IDH mutation status were also utilized in our study.

A total of three models were built to evaluate and compare the performance of the models and parameters. In ‘Model 1’, both MR images and clinical parameters were incorporated into the CNN-LSTM structure. All the nine axial images were passed through each three CNN layer that contains 2 × 2 kernels to create 64, 128, and 256 filters. The binary cross-entropy loss function was minimized using the classical stochastic gradient descent optimizer^23^ at a learning rate of 0.001. ReLu nonlinear function was applied at every CNN layer. Batch normalization and max pooling with 2 × 2 kernel size were applied after every CNN layer. The flatten layer was added at the end of the CNN layers, and the nine flattened patches entered LSTM sequentially. Clinical factors were passed into two successive fully connected layers with four nodes, which were activated by the ReLu function. The outputs of LSTM and the fully connected layer were merged by concatenation. Finally, the merged information was connected to the output of the fully connected layer with one node activated by sigmoid function to determine PsPD or PD.

**Figure 1:**
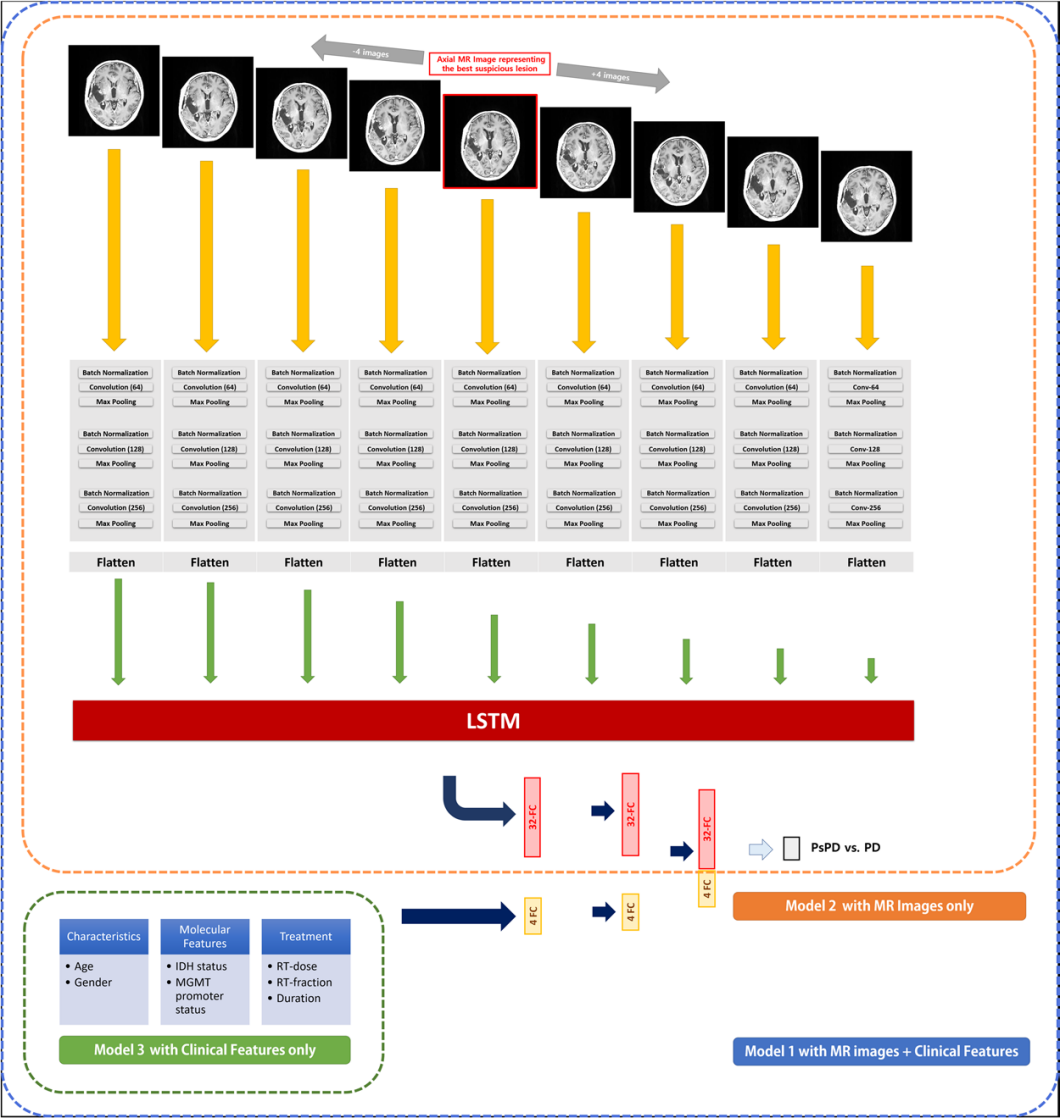
Schematic representation of the structures of the three models. In Model 1 (blue), selected 9 axial MR images were passed through CNN-LSTM, and clinical features were passed into two FCs with four nodes. In Model 2 (orange), only selected 9 axial MR images passed were passed through CNN-LSTM. In Model 3 (green), random forest model using only clinical factors were used. The output is a probability corresponding to PsPD or PD. The number in parentheses in ‘Convolution’ layer means the number of filters made by 2 × 2 pixels kernel. Abbreviations: LSTM, long short-term memory; ML, machine learning; FC, fully-connected layer; PD, progressive disease; PsPD, pseudoprogression; MGMT, O6-methylguanine-DNA-methyltransferase; IDH, isocitrate dehydrogenase.

‘Model 2’ and ‘Model 3’ were developed as a benchmark against ‘Model 1’. In Model 2, MR images but not clinical parameters were used as input of the CNN-LSTM structure. The structure of ‘Model 2’ was identical to that of ‘Model 1’, except the layers of clinical parameters. ‘Model 3’ was built with random forest (RF) classifier to evaluate the ability of clinical parameters without MR images. The number of trees used to train was 1,000 and number of variables randomly sampled at each split was 2. The schematic representation of the structures of the three models is shown in Fig. 1.

Deep learning architecture was implemented using “Keras” wrapper library version 2.0.8 in Python version 3.3 environment with Tensorflow version 1.4 as backend.

### Analysis

Patients collected from SNUH (N = 59) and SNUBH (N = 19) were allocated to training and testing sets, respectively. Because the distribution of binary cases was not uniform, we estimated the area under the ROC curve (AUC) and the area under the precision-recall curve (AUPRC) values to evaluate the trained model in the testing set. Furthermore, we generated the confusion matrix and estimated the precision, recall, and F1-score to compare the performance among three models^24^.

## Results

### Patient Characteristics and Treatment

The clinical characteristics of study patients are presented in Table 1. Thirty (38.5%) and 48 (61.5%) of the CE lesions were PsPD and PD, respectively. There was no significant difference between training and testing sets, except IDH mutation status (p = 0.04, Fisher’s exact test). Female patients tended to present PD rather than PsPD (p = 0.03, Chi-squared test), and the interval between CCRT and CE appearance was significantly shorter in PsPD than PD (p = 0.02, t-test).

**Table 1:** Patient Characteristics.

Variables	PD (N = 48)		PsPD (N = 30)		P-value	SNUH (N = 59)		SNUBH (N = 19)		P-value	Total (N = 78)
	N	%	N	%		N	%	N	%		
Age (median, range)	55.5 (28–77)		55.5 (22–75)		0.95^*^	56 (22–77)		53 (28–75)		0.77^*^	55.5 (22–77)
Gender					0.03^†^					0.18^†^	
Male	27	56.3	24	80		41	69.5	10	52.6		51
Female	21	43.8	6	20		18	30.5	9	47.4		27
MGMT promoter status					0.28^†^					0.61^†^	
Methylated	18	37.5	15	50		24	40.7	9	47.4		33
Unmethylated	30	62.5	15	50		35	59.3	10	52.6		45
IDH status					0.73^‡^					0.04^‡^	
Mutated	1	2.1	2	6.7		3	5.1	0	0		3
Wild-type	43	89.6	26	86.7		54	91.5	15	78.9		69
Unknown	4	8.3	2	6.7		2	3.4	4	21.1		6
Dose schedule of RT					0.80^‡^					0.52^‡^	
Hypofractionated	4	8.3	2	6.7		5	8.5	1	5.3		6
Conventional	44	91.7	28	93.3		54	91.5	18	94.7		72
CE lesion										0.71^†^	
PD						37	62.7	11	57.9		48
PsPD						22	37.3	8	42.1		30
Days to appear of CE lesion (median, range)	123.5 (18–1251)		82 (26–516)		0.02^*^	111 (22–1251)		80 (18–464)		0.91^*^	104 (22–1251)

*Student’s T-test, ^†^Chi-squared test, ^‡^Fisher’s exact test.

Abbreviations: PD, progressive disease; PsPD, pseudoprogression; MGMT, O6-methylguanine-DNA-methyltransferase; IDH, isocitrate dehydrogenase; CE, contrast enhancement; RT, radiation therapy; SNUH, Seoul National University Hospital; SNUBH, Seoul National University Bundang Hospital.

### Negative Control

To identify negative control considering class imbalance, we performed 10-fold internal validation in the scrambled training set. The resulted mean AUC value was 0.47 (Supplementary Fig. S1A). Next, we trained the Model 1 with scrambled training set and tested the finalized model in the testing set (N = 19). The estimated AUC value was approximately 0.5 (Supplementary Fig. S1B). Thus, we considered the ‘luck’ as which results AUC of 0.5 in our downstream analysis.

### Parameter Tuning

**Figure 2:**
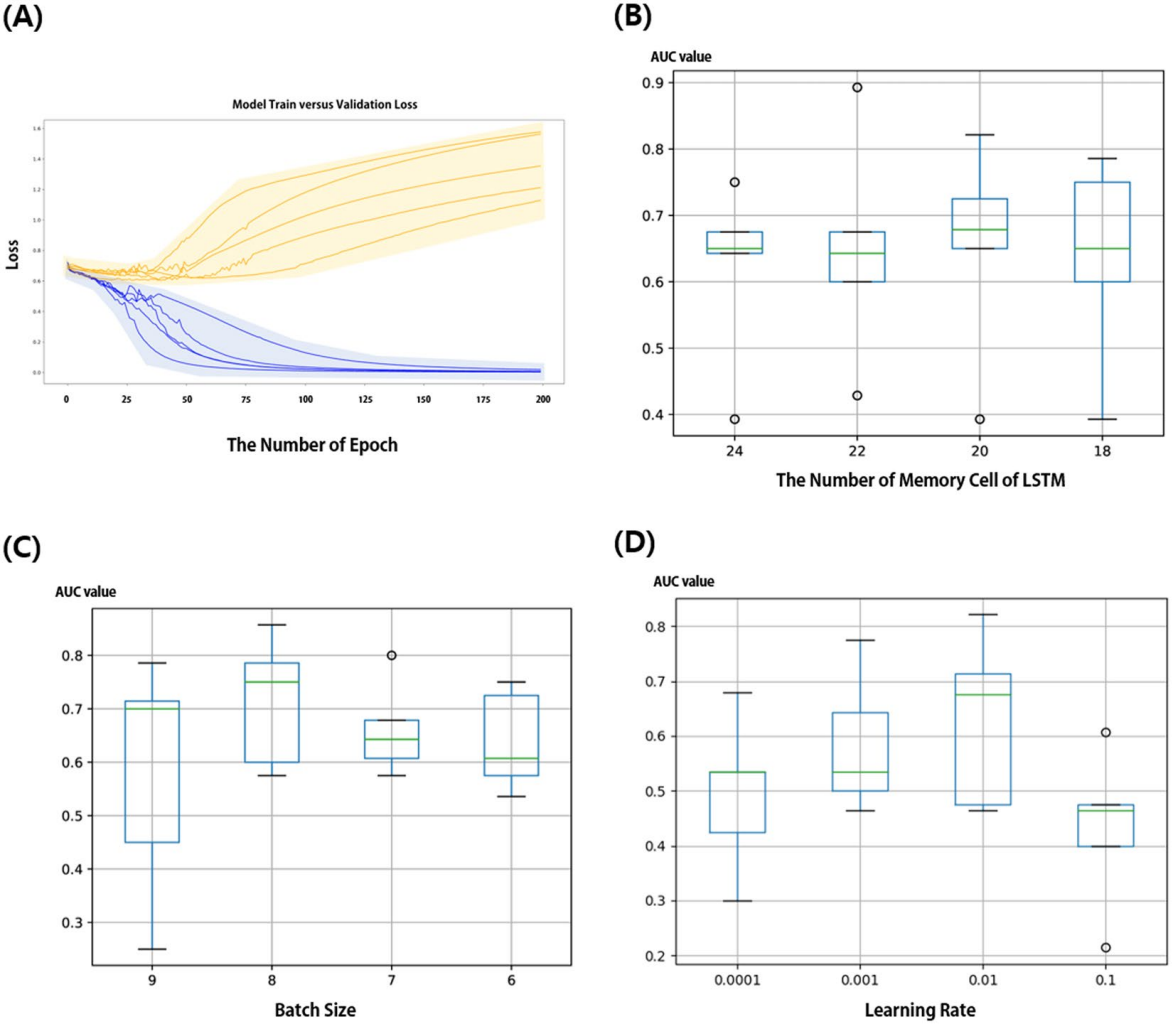
Parameter tuning process. In the training set (N = 59), 20 percent was separated into validation sets. (**A**) Tracing plot representing both train (blue lines) and validation loss (orange lines) through five iterations when learning rate was 0.001, the number of memory cells in LSTM was 24, and batch size was 7. Box and whisker plots depict statistics collected from 5-fold validation, comparing AUC values when (**B**) memory cell size of LSTM was 18, 20, 22, or 24 (**C**) batch size was 6, 7, 8 or 9, and (**D**) learning rate was 0.0001, 0.001, 0.01 and 0.1.

We tuned parameters including the number of epochs, batch size, memory cell size of LSTM, and learning rate of Model 1. First, we sought to find the optimal the number of epochs. We traced the performances of the model in the training set along with the number of epochs in five iterations, plotting train and validation loss when learning rate was 0.001, the number of memory cells in LSTM was 24, and batch size was 8. Twenty percent of the training set was used as the validation set. We assumed that train loss and validation loss would meet at the optimal epoch number. In current study, we found that 25 was the adequate number of epochs to train the model. Figure 2A shows the train and validation traces from each epoch, representing the behavior of the model over time.

Subsequently, we determined the memory cell size by comparing AUC value from 5-fold validation in case of the memory cell sizes of 18, 20, 22, and 24. The statistics of AUC value for each memory cell size are shown in box and whisker plots (Fig. 2B); we selected the most appropriate memory cell size to be 24. To determine the optimal batch size, similar comparison was performed. The batch size was adjusted not to exceed 9 to avoid errors from memory shortage. On the other hand, if the batch size was below 6, training time was significantly long. Therefore, we compared AUC values with batch sizes of 6, 7, 8, and 9 (Fig. 2C); batch size of 8 resulted in the highest AUC value and determined as the optimal value. Lastly, appropriate learning rate was determined to be 0.01 by comparing results from various learning rates which were 0.0001, 0.001, 0.01, and 0.1 (Fig. 2D). The tuned parameters were applied to both Model 1 and Model 2.

### Training

**Figure 3:**
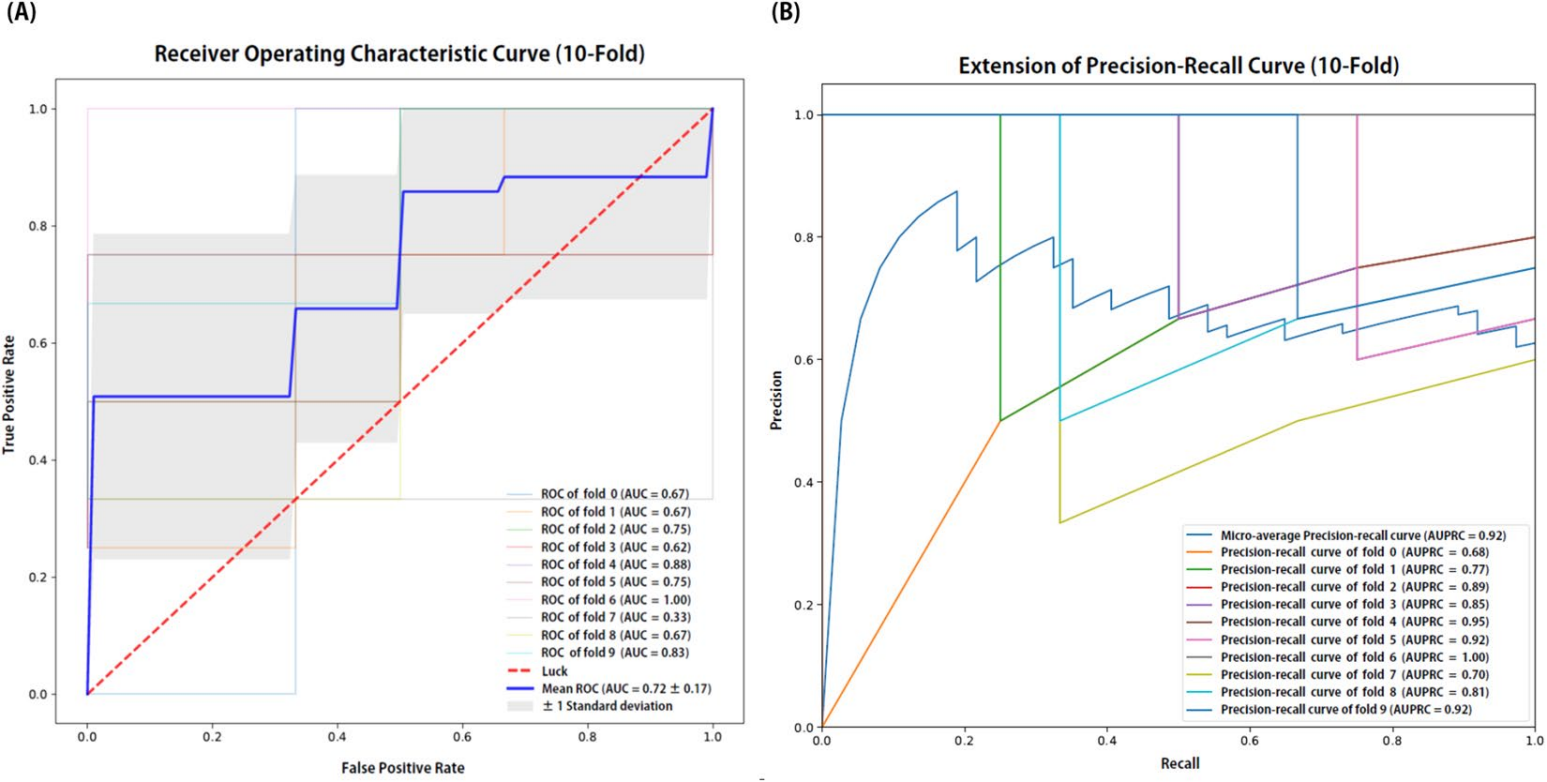
Receiver operating characteristic (ROC) and precision-recall curves from 10-fold internal validation in the training set (N = 59). Area under the ROC curve (AUC) and area under the precision-recall curve (AUPRC) values were also estimated in each fold and represented in graphs. Abbreviations: AUC, area under the curve of ROC curve; AUPRC, area under the precision-recall curve.

We performed a 10-fold cross validation in the training set. ROC curve and precision-recall curve of each fold in Model 1 are shown in Fig. 3A,B, respectively. The estimated values of mean AUC and micro average AUPRC were 0.72 and 0.92, respectively. Approximately 15 minutes to perform 10-fold validation in the training set and 2 minutes to finalize the model were required with 11GB Geforce 1080Ti GPU. Training procedure of Model 2 was same as Model 1. Model 3 was trained in RF classifier with parameters described above.

### Testing and Benchmarking

**Figure 4:**
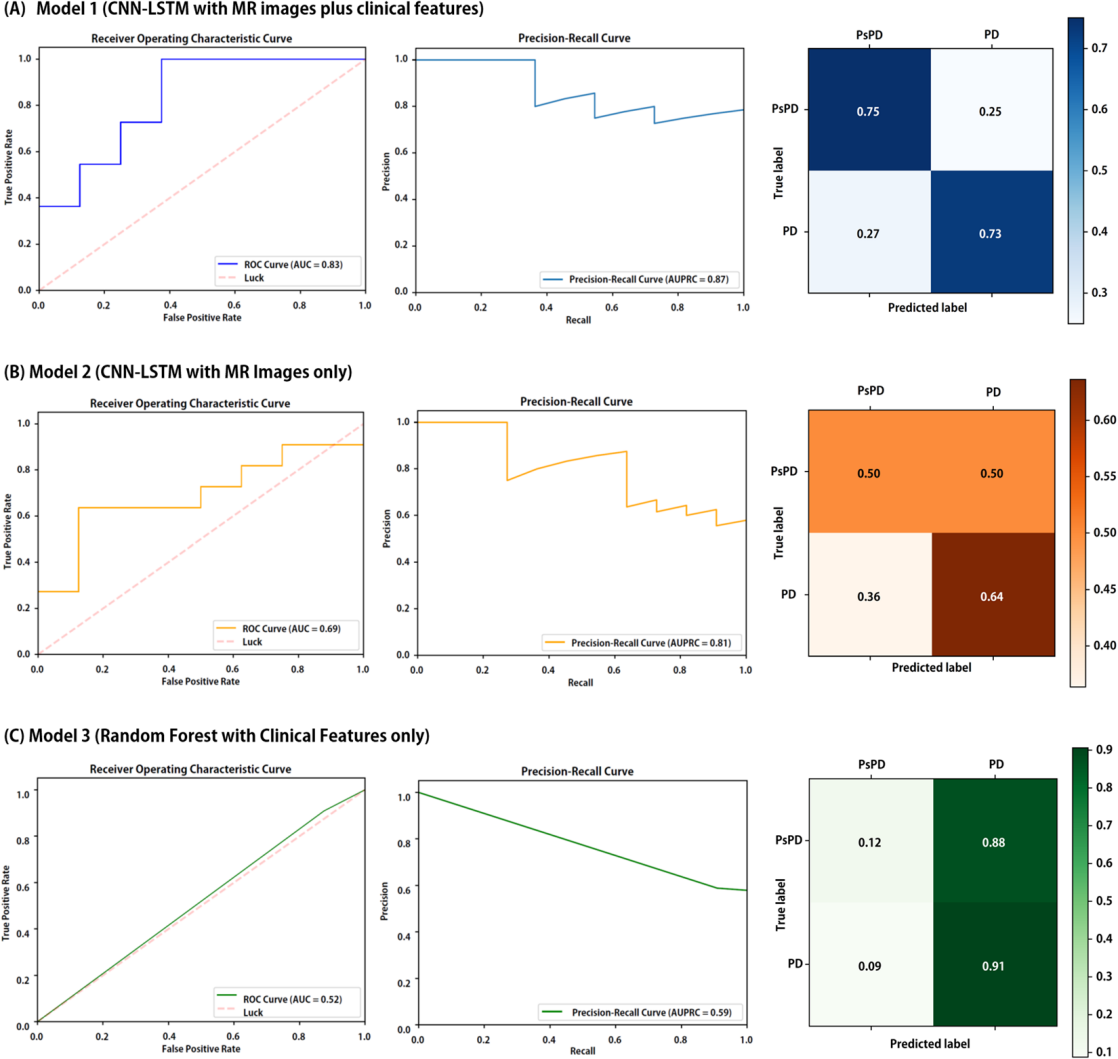
Summarized testing results comparing finalized (**A**) Model 1, (**B**) Model 2, and (**C**) Model 3 in the testing set (N = 19). Receiver operating characteristic curves (left) and precision-recall curves (middle) were depicted and area under the ROC curve (AUC) and area under the precision-recall curve (AUPRC) values were estimated. Normalized confusion matrix (right) also derived. The diagonal number denotes the normalized number of cases where the predicted label is equal to true label. Abbreviations: PD, progression; PsPD, pseudoprogression; AUC, area under the curve of ROC curve; AUPRC, area under the precision-recall curve.

The ROC curve, precision-recall curve, and the normalized confusion matrix of Model 1 in the testing set are depicted in Fig. 4A. The estimated values of AUC and AUPRC were 0.83 and 0.87 in testing set, respectively. The optimal threshold value was determined when the true positive rate (TPR) was high and the difference of TPR and (1-false positive rate) was nearly zero. As a result, the average precision, average recall, and average F1-score of ‘Model 1’ were 0.74, 0.74, and 0.74, respectively.

The results of Model 2 in the testing set is demonstrated in Fig. 4B. The average precision, average recall, and average F1-score of this model were 0.58, 0.58, and 0.85, respectively. The estimated values of AUC and AUPRC was 0.69 and 0.81, respectively. As shown in Fig. 4C, the estimated values of AUC and AUPRC of Model 3 were 0.52 and 0.59, respectively, indicating that the performance was the best in Model 1. Results of all models are summarized in Table 2.

**Table 2:** Summary of Result and Model Comparison.

Model 1		Precision	Recall	F1-score
CNN-LSTM with MR images and clinical information	PD	0.67	0.75	0.71
	PsPD	0.80	0.73	0.76
	Average	0.74	0.74	0.74
**Model 2**		**Precision**	**Recall**	**F1-score**
CNN-LSTM with MR images only	PD	0.50	0.50	0.50
	PsPD	0.64	0.64	0.64
	Average	0.58	0.58	0.58
**Model 3**		**Precision**	**Recall**	**F1-score**
Random Forest with clinical information only	PD	0.00	0.00	0.00
	PsPD	0.56	0.91	0.69
	Average	0.32	0.53	0.40

Abbreviations: PD, progressive disease; PsPD, pseudoprogression; CNN, convolutional neural network; LSTM, long short-term memory.

## Discussion

In current study, we presented a novel ML algorithm to predict PsPD in GBM patients after adjuvant CCRT, given both MRI showing a suspicious CE lesion and clinical factors. Our algorithm achieved a moderate predictability in the unseen testing set (AUC = 0.83, AUPRC = 0.87, and F1-score = 0.74) collected from the independent institution. To our knowledge, the present study is the first to use deep ML algorithm for the identification of PsPD in GBM patients.

Early differentiation between PsPD and PD is extremely important in terms of salvage treatment. Many researchers have investigated the usefulness of radiologic features for the prediction. Several authors have reported that diffusion and perfusion MRI have additional roles in detecting PsPD. By combining parameters from diffusion tensor imaging and perfusion imaging, Wang *et al*. built a model differentiating PsPD from non-PsPD with AUC of 0.807^25^. Prager *et al*. also demonstrated a model using diffusion and perfusion MRI that yielded 93.1% sensitivity and 83.3% specificity in predicting PD^26^. Recent meta-analysis revealed that PET provided better accuracy in detecting recurrent tumors than conventional MRI^27^. Despite the role of diffusion and perfusion images, there are still no specific guideline involving the advanced imaging modalities^22^. Therefore, the model using the conventional MRI may have potency in terms of widespread use and easy validation. Chen *et al*.^28^ attempted to predict PsPD using texture features of T1-weighted and T2-weighted MRI and showed an accuracy of 86.4% using the model, suggesting the potential role of conventional images. However, they included 22 patients and did not validate the model.

Recently, ML algorithms have been an attractive tool in analyzing medical images. Several investigators utilized support vector machine classifier and multi-parametric MR images^29,30^, but the results were not externally validated. The *deep* and *sequential* ML algorithm adopted in our analysis is widely used. CNN has been utilized for segmentation or classification of brain tumor on MR image^31–35^. We avoided to use modern CNN models, such as ‘GoogLeNet’^36^, because deeper structures may cause worse performance with small number of samples. Nevertheless, the F1-score of our algorithm was acceptable in external validation.

One of the strengths of our study is incorporation of clinical parameters. No previous ML studies included clinical factors in their models. It is well known that some clinical variables are associated with the likelihood of PD or PsPD. For instance, methylation of MGMT and IDH mutation is associated with the formation of PsPD and disease progression^37,38^. Consequently, we hypothesized that clinical parameters could improve the performance of ML model, and the hypothesis was tested by developing the three models. Our results suggest that model including both MR and clinical features performs better than models including only one of them. Given that CNN-LSTM is a black-box technique, we cannot quantify exact importance of features among input data. However, it implies that combining radiologic and clinical data in discriminating PsPD is necessary not only in clinic but also in future investigations. Minimization of intervention by clinician is another advantage of our models where CE lesions were not needed to be segmented. The segmentation process is subjective to operator, labor-intensive, and difficult to be automated.

We selected 9 axial images to be incorporated in the models. The number of input images depends on training resources such as data availability, time, cost, or algorithm. Nine images are easy to handle and make developers use moderate-scale algorithm without expensive high-end computers with less time to train and predict. Moreover, using whole MR image set may cause ‘curse of dimensionality’^39^ because the number of samples is small and could possibly consume more computational resources. On the other hand, we utilized MR images with only enhanced T1-weighted sequence. The MPRAGE sequence is widely used in daily practice and can be acquired with both 1.5-T and 3-T MR scanners, facilitating validation and application of the developed algorithm^40^. Furthermore, including other sequences may also increase the input dimension compared to the sample size. Eventually, 9 input images with one sequence were selected as input for the algorithm, which is acceptable given the sample size and the clinical applicability of the model.

Difficulty in defining PD and PsPD is an intrinsic limitation of the study. While only surgical resection can confirm the identity of lesion, the majority of patients (70.5%, 55/78) did not receive the second resection due to poor performance of patients. Instead, we used strict criteria in discriminating PD and PsPD, which was in concordance with multi-disciplinary decision.

Compared to other ML studies dealing with a binary decision problem, however, metrics estimated from the present study appears not remarkable. Small number of cases was one of the possible reasons. Basically, the incidence of GBM is low and the inclusion criteria of the study were strict. We excluded patients who received partial resection of tumor because they cannot exhibit pure PsPD. Those with short follow-up period were also excluded. Due to small datasets, our model could have been overfitted; hence, further validation with more samples is required to confirm the clinical utility of our model.

In addition, one may consider adversarial examples, which were recently reported to attack deep neural network^41^, in our study. Existence of adversarial examples and defense against them are under investigation. Concerning the existence of adversarial examples, which might lead to false decision, Szegedy *et al*.^42^ addressed that the probability of adversarial examples is extremely low and they are hardly seen in testing sets. Another study^43^ reported that adversarial examples are mainly distributed with low probability compared to clean data. Nevertheless, we attempted to generate adversarial images with fast gradient sign method^44^, which adds perturbation to original images. However, perturbation cannot be simply calculated from backpropagation since our CNN is involved with LSTM and neural network taking clinical features. Most adversarial cases focus only on image classification using deep neural network and, to our knowledge, there is no adversarial examples about CNN-LSTM model to date. If adversarial examples of our model exist and can be found in future studies, the performance of our model would be improved.

In conclusion, we developed a deep ML algorithm that can be applied to differentiate PsPD and PD in GBM patients who had completed current standard therapy. With 9 selected axial MR images and clinical factors, the model showed acceptable performance in the independent dataset. Our algorithm could help making decision during follow-up. Further validation studies with larger samples from various institutions is necessary to ensure the clinical utility of this model.

## Electronic supplementary material

Supplementary Information

## Data Availability

The Python source codes of the process are free and available at https://github.com/bigwiz83/PsPDvsPD. However, the analyzed datasets cannot be opened publicly due to the law for handling of medical information in Korea. Reasonable request following approval from institutional review board is required to access to the datasets.
