# Supplementary material for: Prediction of Pseudoprogression versus Progression using Machine Learning Algorithm in Glioblastoma
Source: Sci Rep. 2018 Aug 21;8:12516. doi: 10.1038/s41598-018-31007-2 (PMC6104063; doi:10.1038/s41598-018-31007-2)
Supplement: Supplementary file 1 — Supplementary Information [file 41598_2018_31007_MOESM1_ESM.pdf]

Supplementary Information

Title: Prediction of Pseudoprogression versus Progression using Machine Learning Algorithm in Glioblastoma

Authors: Bum-Sup Jang[1*], Seung Hyuck Jeon[1*], Il Han Kim[1,3] and In Ah Kim[2,3]

*These authors contributed equally to this work.

Affiliations:

[1]Department of Radiation Oncology, Seoul National University Hospital, Seoul, Republic of Korea

[2]Department of Radiation Oncology, Seoul National University Bundang Hospital, Seongnamsi, Korea

[3]Institute of Radiation Medicine, Cancer Research Institute, Seoul National University College of Medicine, Seoul, Korea.

Supplementary Figure S1. (A) Ten-fold validation of model with scrambled training set, and (B) the resulted ROC curve in the testing set. The result suggests that scrambled inputs plays a negative control since AUC are close to 0.5. Abbreviations: AUC, area under the curve of ROC curve

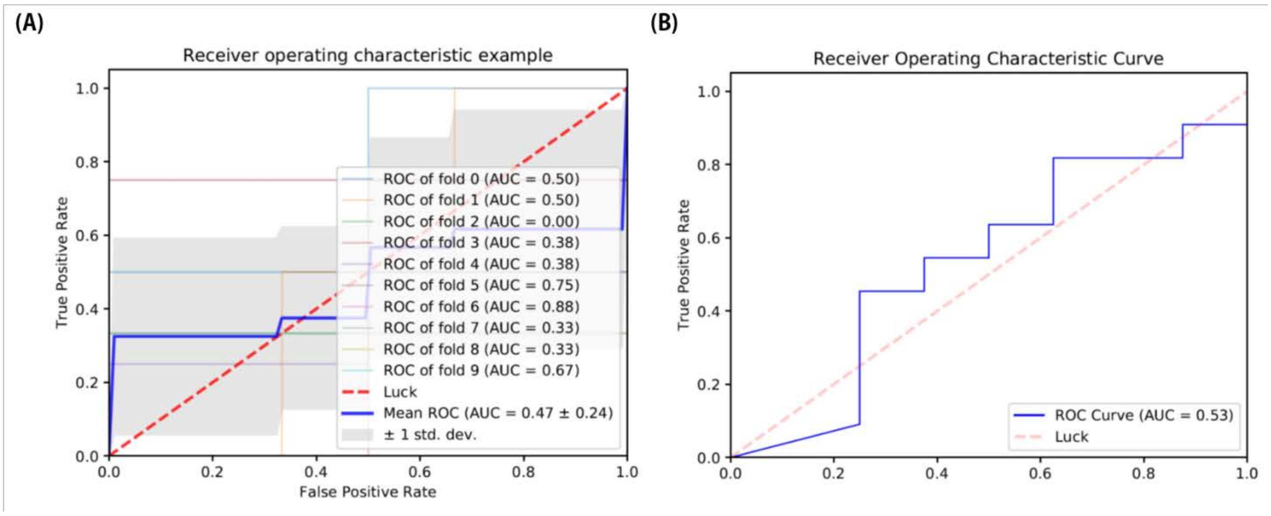

Supplementary Table S1. Properties of obtained MR images included in the analysis.

		Training set (N=59)	Testing set (N=19)
Magnetic field	1.5-T	11 (18.6%)	0 (0.0%)
	3-T	48 (81.4%)	19 (100.0%)
Pixel Matrix	256 x 256	33 (55.9%)	0 (0.0%)
	480 x 480	6 (10.2%)	8 (42.1%)
	512 x 512	17 (28.8%)	10 (52.6%)
	560 x 560	0 (0.0%)	1 (5.3%)
	1024 x 1024	3 (5.1%)	0 (0.0%)
Slice thickness (mm)		1.0 (0.86-1.5)	1.0 (1.0-1.0)
Axial pixel width (mm)		0.45 (0.38-0.50)	0.98 (0.23-1.0)
Field of view (mm)		250 (200-256)	226 (193-256)
Repetition time (msec)		1500 (7.968-2100)	8.204 (8.103-500)
Echo time (msec)		1.9 (1.88-4.72)	4.605 (4.605-34.15)
Flip angle (degree)		9 (8-20)	8 (8-90)

Abbreviations: T, tesla, MR, magnetic resonance
